# Degenerative joint disease: from a conservative to a minimally invasive approach – clinical case

**DOI:** 10.1080/07853890.2021.1897368

**Published:** 2021-09-28

**Authors:** Mariana Alberto, Pedro Cebola, João Rua, Maria Braz de Oliveira, André Maria de Almeida, José João Mendes

**Affiliations:** a Instituto Universitário Egas Moniz (IUEM), Egas Moniz Cooperativa de Ensino Superior, Caparica, Portugal; b Centro de Investigação Interdisciplinar Egas Moniz (CiiEM), Egas Moniz Cooperativa de Ensino Superior, Monte de Caparica, Caparica, Portugal

## Abstract

**Introduction:**

Degenerative joint disease is a subtype of temporomandibular derangements. It can be characterised by possible progressive cartilage degradation, subchondral bone remodelling, and chronic inflammation in the synovial tissue [1]. For the treatment of temporomandibular disorders, minimally invasive techniques, such as viscosupplementation, should normally be applied after more conservative techniques [2].

**Materials and methods:**

Female patient, 22 years old, with probable disc displacement with occasional blocking and bilateral degenerative joint disease. Before starting any treatment, the patient was asked to sign an informed consent. Diagnosis was made through the symptom questionnaire and clinical exam integrated on the Diagnostic Criteria for Temporomandibular Disorders (DC/TMD) protocol [3] along with imagiological exams. Definitive diagnosis was disc displacement with occasional blocking, bilateral degenerative joint disease, bilateral arthralgia, masseter myalgia and bruxism self-report. On a first approach we did behavioural and cognitive therapy, viscosupplementation with hyaluronic acid 1.5% of high molecular weight (Syaloset 2000^®^), anterior repositioning splint drawn and printed with CAD-CAM technique (Exocad dentalCAD^®^) and physiotherapy exercises, like manual therapy for muscular relaxation and condylar distraction, as well as, at-home physiotherapy exercises. For evaluate if there were any improvement on function, comfort and quality of life the OHIP-14 questionnaire [4] was applied before and after the viscosupplementation and again, after 1 month of the occlusal splint use. The same thing was done with the DC/TMD protocol.

**Results:**

According to the OHIP-14 questionnaire, before treatment the patient mainly referred the articular pain that she felt during mastication. After the first viscosupplementation session and the new OHIP-14 questionnaire application, the patient referred substantial improvements on the pain during function. However, she expressed her concerns with the expectations of the treatment. After 1 month of occlusal splint use her concerns were gone and pain during function did not come back.

**Discussion and conclusions:**

Disc displacements should be carefully evaluated before the prescription of an anterior repositioning splint. However, in cases like this, they are indicated [5]. In this case, viscosupplementation was used in the beginning of treatment to allow the TMJ to recover function without limitations and restoring a dynamic lubrification. Treatment of TMJ disfunctions should be conservative and always multidisciplinary.

## References

[1] Wang X, Zhang J, Gan Y, et al. Current understanding of pathogenesis and treatment of TMJ osteoarthritis. J Dent Res. 2015;94(5):666–673. 25744069 10.1177/0022034515574770

[2] Januzzi E, Ferreira LA, Grossmann E, et al. Effectiveness of sequential viscosupplementation in temporomandibular joint internal derangements and symptomatology: a case series. Pain Res Manage. 2018;2018:1–9. 10.1155/2018/5392538 PMC6091395 30154944

[3] Schiffman E, Ohrbach R, Truelove E, et al. Diagnostic criteria for temporomandibular disorders (DC/TMD) for clinical and research applications: recommendations of the International RDC/TMD Consortium Network and Orofacial Pain Special Interest Group. J Oral Facial Pain Headache. 2014;28(1):6–27. 24482784 10.11607/jop.1151 PMC4478082

[4] Amaral JA, Marques D, Godinho J, et al. Validação de uma versão portuguesa do questionário Oral Health Impact Profile‐14. Revista Portuguesa de Estomatologia, Medicina Dentária e Cirurgia Maxilofacial. 2014;7:55.

[5] Greene CS, Menchel HF. The Use of Oral Appliances in the Management of Temporomandibular Disorders. Oral Maxillofac Surg Clin North Am. 2018;30(3):265–277. 29866449 10.1016/j.coms.2018.04.003

